# Vesicular Disease in 9-Week-Old Pigs Experimentally Infected with Senecavirus A

**DOI:** 10.3201/eid2207.151863

**Published:** 2016-07

**Authors:** Nestor Montiel, Alexandra Buckley, Baoqing Guo, Vikas Kulshreshtha, Albert VanGeelen, Hai Hoang, Christopher Rademacher, Kyoung-Jin Yoon, Kelly Lager

**Affiliations:** US Department of Agriculture, Ames, Iowa, USA (N. Montiel, A. Buckley, V. Kulshreshtha, A. VanGeelen, K. Lager);; Iowa State University College of Veterinary Medicine, Ames (B. Guo, H. Hoang, C. Rademacher, K.-J. Yoon)

**Keywords:** Senecavirus A, Seneca Valley virus, viruses, nasal infection, vesicular disease, experimental infections, pigs

## Abstract

Senecavirus A has been infrequently associated with vesicular disease in swine since 1988. However, clinical disease has not been reproduced after experimental infection with this virus. We report vesicular disease in 9-week-old pigs after Sencavirus A infection by the intranasal route under experimental conditions.

Senecavirus A (SVA), formerly known as Seneca Valley virus, is a nonenveloped, single-stranded, positive-sense RNA virus that belongs to the family *Picornaviridae* and has recently been proposed to be the prototype species of the genus *Senecavirus* (*1*). Although SVA was first identified as a contaminant in cell culture medium (*2**,**3*), it has been infrequently associated with cases of idiopathic vesicular disease in pigs in the United States (*3**,**4*) and Canada (*5*). These findings have led to speculation that SVA infection could be confused with a highly contagious vesicular livestock disease caused by foot-and-mouth disease virus (FMDV), another picornavirus in the genus *Apthovirus*. SVA infection has rarely been reported in other countries.

Beginning in late 2014, vesicular disease was reported in many swine herds in Brazil, and SVA was identified in serum, vesicular fluid, and swab samples from ruptured vesicles collected from affected weaned and adult pigs (*6**, **7*). In July 2015, an unprecedented emergence of vesicular disease began in multiple swine herds in the United States, and only SVA was detected in samples from affected animals. Presumably, SVA is the cause of these current epidemics of vesicular disease in Brazil and the United States. However, a causal relationship between the virus and its host has not been made.

We report vesicular disease in nursery-age pigs that were experimentally infected with an SVA isolate obtained from a commercial swine operation in South Dakota, United States. These pigs had idiopathic vesicular disease with lameness.

## The Study

We purchased 17 conventionally raised weaned pigs and housed them until 9 weeks of age at the campus of the Agricultural Research Service, National Animal Disease Center, US Department of Agriculture (Ames, IA, USA), in accordance with Institutional Animal Care and Use Committee protocols (protocol ACUP 2867). At this time, each pig received an intranasal inoculation of a cell culture–propagated SVA isolate (SVA15–41901SD, third passage) (B. Guo, unpub. data) at a dose of 5 ×10^7^ PFU/animal. Challenge virus was grown in a swine testicular cell line (CRL-1746; American Type Culture Collection, Manassas, VA, USA) and tested for extraneous viruses by using PCRs and next-generation sequencing.

We detected no viruses other than SVA in the challenge inoculum. At 2, 4, 6, 8, and 10 days postinfection (dpi), we euthanized a randomly selected pig and conducted necropsy. Although we used the remaining 12 pigs to evaluate the kinetics of virus infection and euthanized them at 36 dpi for the purposes of this study, we describe only the acute phase of infection here.

We collected blood samples at 0, 3, and 15 dpi and monitored all pigs for vesicular and erosive lesions on the snout and hooves. When we detected vesicular lesions, they were swabbed, and vesicular fluid was collected from intact vesicles. We stored serum harvested from blood samples, swab samples, and vesicular fluid at −80°C until testing for SVA RNA by using a primer- and probe-based, quantitative, real-time, reverse transcription PCR. Serum was tested by using an indirect fluorescent antibody test with human lung cancer cells (CRL-5803; American Type Culture Collection) for detection of antibodies against SVA (B. Guo, unpub. data).

**Figure 1:**
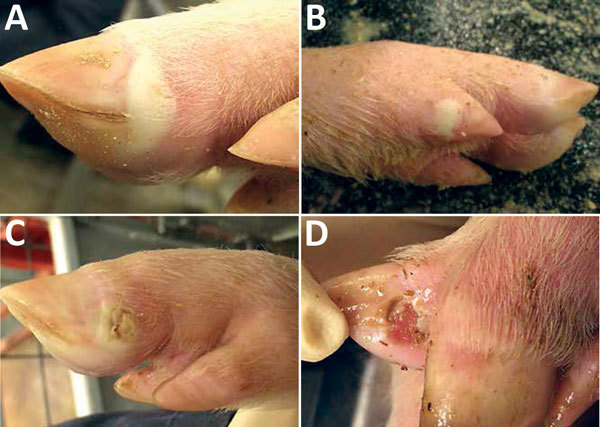
Vesicular lesions on feet of pigs experimentally infected with Senecavirus A. A) Blanched, intact, fluid-filled vesicle on lateral coronary band of toe. B) Intact vesicle on coronary band of medial dewclaw. C) Ruptured vesicle on coronary band of toe. D) Ruptured vesicle with ulceration and erosion in interdigital space.

**Figure 2:**
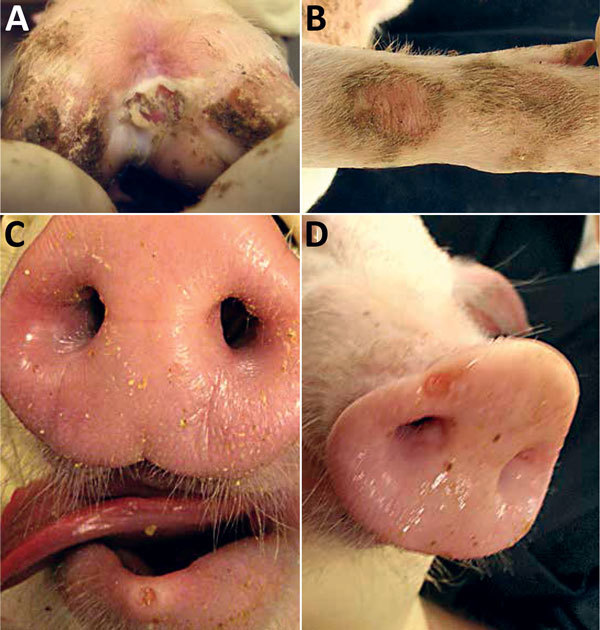
Vesicular and skin lesions on feet and snout of pigs experimentally infected with Senecavirus A. A) Ruptured vesicle with deep ulceration, necrosis, and crusting in interdigital space. B) Skin abrasion on carpus. C) Vesicle and erosion on lower lip. D) Vesicle on snout.

At 4 dpi, 7 of 16 pigs had intact or ruptured vesicular lesions on the coronary bands of toes and dewclaws or the interdigital space of >1 feet (Figure 1). We observed minimal-to-mild lameness in some animals. After 5 dpi, 14 of 15 pigs had new or previously observed vesicular lesions. Lesion severity ranged from blanched coronary bands to ulcerations and erosions from ruptured vesicles. Focal necrosis and crusting of either the interdigital space (Figure 2, panel A), the coronary band, or both developed in some animals. Severe lesions in a subset of the cohort progressed to multifocal deep ulcers. In general, vesicular lesions were 0.2–2 cm in diameter. However, we observed several pigs with skin abrasions over the carpus (Figure 2, panel B), which indicated that the pigs were moving while knuckling, which was probably caused by having tender feet.

At 5–6 dpi, we observed a single, small, rounded vesicle and erosion on the lower lip (Figure 2, panel C) in 2 pigs. After 10 dpi, no new lesions were detected in any of the extremities or lower lips, and old lesions were healing. We first observed vesicular lesions and erosions on snouts only at 10 dpi in the same animals that had vesicular lesions on the feet (Figure 2, panel D). Of the 5 pigs euthanized, vesicular lesions were visible on coronary bands of >1 extremities of pigs subjected to necropsy at 4 and 6 dpi. We observed no other gross abnormalities at necropsy. Water and food consumption was not affected during the course of the disease and no animals died on their own.

Before inoculation, all pig serum samples were negative for SVA RNA and antibody. We detected SVA RNA in serum samples from each pig at 3 dpi (range 1.1 × 10^2^–8 ×10^5^ genomic copies/μL) and in all swab samples collected from pigs with vesicular lesions at 5 dpi (1.9 × 10^1^–7.9 × 10^4^ genomic copies/μL). We also identified SVA by PCR on swab samples from snout ulcers. All surviving pigs seroconverted to SVA by 15 dpi, as determined by indirect fluorescent antibody test (titer >1:640).

## Conclusions

Idiopathic vesicular disease in swine is a diagnosis made when none of the known etiologies for swine vesicular disease (i.e., vesicular exanthema virus, swine vesicular disease virus, vesicular stomatitis virus, and FMDV) have been detected in a clinical case. SVA has been detected occasionally in cases of idiopathic vesicular disease, which increases the possibility that SVA infection could cause vesicular disease in swine. This assumption was strengthened by the recent emergence of idiopathic vesicular disease in Brazil and the United States in which there was common detection of SVA.

In this study, we experimentally induced clinical signs and gross lesions in nursery-age pigs inoculated with SVA, demonstrating a causative relationship between SVA infection and vesicular disease in susceptible pigs. This finding is noteworthy because SVA disease appears to be clinically indistinguishable from other vesicular diseases of swine (*4**,**5**,**8*), especially FMDV (*9**–**11*), which is a highly transmissible livestock disease that can cause devastating economic losses to the agricultural industry and disruption of the human food supply. However, unlike the typical clinical progression of FMD in swine in which feet and snout lesions develop at about the same time, SVA-induced vesicular disease may have a different temporal pattern. In this study, we observed lesions on the feet several days before any lesions were recognized on the snout, which might be related to the route of inoculation or other factors.

We have begun to elucidate the clinical disease and host responses to SVA infection in swine. However, further investigation is needed to address 1) susceptibility of other age groups to this isolate and other SVA isolates; 2) the contribution to disease of co-infection with other infectious agents and stressful conditions, such as transport and heat; and 3) if viral mutations could explain, at least partially, the increase in recent SVA case reports. A better understanding of SVA pathogenesis might help in development of prevention and control measures and differentiation of this virus from those causing other vesicular diseases.
